# Lifestyle Behaviors and Their Socio‐Demographic Correlates Among University‐Going Adolescents in Rawalpindi, Pakistan: A Cross‐Sectional Study

**DOI:** 10.1002/hsr2.73240

**Published:** 2026-09-17

**Authors:** Meesha Rao, Humaira Mahmood, Abdul Momin Rizwan Ahmad, Saira Zafar, Hasnain Khalid, Manaar Adil Siddiqui, Naseem Azad, Attiya Najeeb Abbasi, Nilab Mohammadi

**Affiliations:** ^1^ Department of Research and Development Armed Forces Institute of Cardiology/National Institute of Heart Diseases Rawalpindi Pakistan; ^2^ School of Public Health Health Services Academy Islamabad Pakistan; ^3^ Department of Human Nutrition and Dietetics, NUST School of Health Sciences National University of Sciences and Technology (NUST) Islamabad Pakistan; ^4^ Department of Health Sciences University of York York UK; ^5^ School of Management Xuzhou Medical University Xuzhou China; ^6^ Department of Public Health, Armed Forces Postgraduate Medical Institute (AFPGMI) National University of Medical Sciences (NUMS) Rawalpindi Pakistan; ^7^ Department of Curative Medicine Ghalib University Herat Afghanistan

**Keywords:** adolescents, lifestyle behaviors, physical activity, socio‐demographic correlates, university students

## Abstract

**Objective:**

Adolescence is a critical period where lifestyle habits, including diet, physical exercise, sleep, and substance abuse, may have a significant influence on future health. The objective of this study was to determine adolescents' lifestyle behaviors and how they relate to socio‐demographic characteristics.

**Methods:**

The study included university‐going older adolescents in Rawalpindi, Pakistan and employed a cross‐sectional study design. Participants completed a validated questionnaire assessing lifestyle behaviors and their socio‐demographic correlates, along with anthropometric measurements for BMI categorization. Chi‐square test and multivariate logistic regression were used for analysis.

**Results:**

The present study included 215 university‐going older adolescents (mean age: 18.69 SD = 0.46 years); the majority (75.8%) were female participants, and 70.2% were urban inhabitants. The majority had a healthy BMI (68.8%) and ate three meals each day (90.7%), but less than 12% were physically active. Over a quarter reported not getting enough sleep. 28.4% of participants reported depressed symptoms; just 10.2% addressed their problems with a specialist. 9.8% used drugs (smoking), 20.9% vaped, and 25.6% began using before the age of 12. There were significant (*p* < 0.05) associations between lifestyle behaviors and socio‐demographic characteristics such as age, BMI, and family type. Regression analysis showed BMI, residence, and parental education as key predictors of adolescent lifestyle.

**Conclusion:**

The findings revealed significant gaps in healthy lifestyle behaviors university‐going older adolescents in Rawalpindi.

## 1 Introduction

Adolescence is the critical stage of life for adopting health behaviors like diet, physical activity, sleep, and drug use, which influences long‐term risk for non‐communicable diseases. The present study seeks to focus on older adolescents (18–19) as they transition to postsecondary education autonomy and exposure to new lifestyle hazards [1]. Adolescents encounter rapid development in their bodies, feelings, and lifestyles, and this is when they begin to discover their personalities and excellent health practices [2]. Adolescents are expected to number over 1.3 billion worldwide, accounting for roughly 16% of the total population [3]. Adolescence is regarded as the second most important stage of human development after infancy, because it is characterized by rapid growth.

The great majority of teens reside in low‐ and middle‐income nations, and their numbers are predicted to increase by 2050 [4]. The everyday routines of the adolescent age group can have a significant impact on their current and future health [5]. Environment and background can impact an individual's likelihood of having NCDs in adulthood [6]. Adolescents' health is often overlooked in global health programs [7]. The Lancet Commission notes that many adolescents are not getting enough nutrients, describing it as a “hidden catastrophe” [7], since programs fail to account for their growing demands [8].

In Pakistan, there is insufficient evidence on the types and extent of behaviors‐ such as physical activity, nutrition, mental well‐being, and substance use‐ that define adolescent lifestyles. This issue is most important throughout adolescence because NCDs and mental health problems are on the rise and can be controlled if addressed promptly, and good habits are formed [9, 10]. Primordial prevention, which focuses on preventing the development of risk factors for disease, is particularly important in adolescence, as during this stage, habits related to food and exercise are established [11, 12]. Maladaptive behaviors during adolescence, such as a bad diet and physical inactivity, are powerful contributors to the development of NCDs (Tayyem et al., 2022 [13]).

Working on these issues is consistent with the United Nations' SDGs (Sustainable development goals), particularly SDG 3 (Good Health and Well‐being), SDG 1 (No Poverty), and SDG 4 (Quality Education). SDG Target 3.4 aims to reduce NCD (Non‐communicable disease)‐related premature deaths by one‐third by 2030, primarily by prevention and treatment. The objective of this study is to assess the lifestyle behaviors of university‐going adolescents in Rawalpindi, Pakistan, and to examine their association with socio‐demographic factors.

## 2 Methods

### 2.1 Study Design and Objective

The present study used a quantitative cross‐sectional research design to evaluate adolescents' lifestyle behaviors and how they relate to socio‐demographic characteristics. The study was conducted at a public university in Rawalpindi, Pakistan.

### 2.2 Study Settings

The study was conducted at a public sector university, Rawalpindi, a multi‐faculty undergraduate institute, during the time period of September 2024 to January 2025.

### 2.3 Study Population

The eligible participants consisted of older adolescents aged 18 to 19 who were already attending a university in Rawalpindi.

### 2.4 Sampling Technique

The study used a purposive sampling strategy to efficiently recruit the older adolescents aged 18–19 available on the campus. Purposive (non‐probability sampling) enables concentrated recruitment but may impose selection biases and restrictions. Generalizability outside similar university populations.

### 2.5 Sample Size Estimation

The required sample size was determined using the WHO sample size calculator, assuming a prevalence of metabolic syndrome among adolescents as 16.7% (Sharif et al., 2024), 95% confidence interval, and 5% margin of error. The calculated minimum sample size was 215 participants. As no directly comparable prevalence estimate for adolescent lifestyle behavior was available in the Pakistani literature, metabolic syndrome prevalence was used as the closest available reference for sample size estimation.

### 2.6 Operational Definitions

#### 2.6.1 Current Smoker

Someone who reports smoking at least one cigarette within last 30 days.

#### 2.6.2 Current Vaper

Someone who reports use of any e‐cigarettes in last 30 days.

#### 2.6.3 Early Initiation of Smoking or Vaping

First use before the age of 12.

#### 2.6.4 Inadequate Sleep

Sleeping less than 8 h or after midnight.

#### 2.6.5 Physical Activity

Engaging in any form of exercise or physical movement more than three times a week or reporting that it is a regular part of their routine.

#### 2.6.6 Depressive Symptoms

Reporting of affirmative response of experiencing feelings of helplessness, worthlessness, or hopelessness.

### 2.7 Data Collection Tools

A well‐designed and thoroughly pre‐tested Adolescent Lifestyle Questionnaire based on the items developed by Qidwai et al. 2010 [14]. It covered socio‐demographic data, diet, hydration, Sleep patterns, screen time, Physical activity and bullying, Substance use (smoking/vaping), and Mental health indicators. In addition, the Kuppuswamy Socioeconomic Scale was used to categorize participants' socioeconomic position based on their schooling background, occupation, and household income. The instrument was pilot tested on 20 students for clarity and face validity. The enumerator administered anthropometry and supervised self‐completion of questionnaire.

Validity and reliability of instrument: the items of questionnaire were derived from published study of [14]. And pilot tested for face validity. Full psychometric data were not available for adapted single item measures, which is acknowledged in limitation.

#### 2.7.1 Anthropometry and BMI

height and weight were measured to nearest 0.1 cm and 0.1 kg using portable stadiometer and calibrated digital scale respectively, then BMI was calculated as weight (kg)/height (m^2^) and was categorized using WHO cut off: underweight < 18.5, normal 18.5–24.9, overweight 25–29.9, obese> 30.

### 2.8 Variables of Interest

The questionnaire included questions about their lifestyle (what they eat, how much water they drink, how often they exercise, sleep, use substances, and their mental health), as well as their age, gender, BMI, parental education, and their own living situation.

### 2.9 Data Collection

Self‐administered questionnaires under the supervision of enumerators were used to obtain the data, as the participants completed the survey on‐site, the researcher was able to get accurate answers and understandings from them. Of the 524 students approached during the study period, 309 declined to participate and rest were enrolled to complete the required sample of 215 participants

Lifestyle: lifestyle domains were assessed across five domains derived from the adapted adolescent lifestyle questionnaire by [14]; the scoring system was developed conceptually based on existing literature on adolescent lifestyle behavior and reviewed by two domain experts for content validity. Each domain was scored differently to reflect healthy or risky behavior. The domains were: water intake (adequate hydration was defined as > or equal to 8 glasses of water per day, with a score of 1. Inadequate hydration was defined as less than 8 glasses of water, scored as 0. Healthy physical activity was defined as engaging in any form of exercise or physical movement more than three times a week or reporting that it is a regular part of their routine, with a score of 1, and physical exercise not being a part of routine was scored as 0. With regards to dietary habits, reporting of eating just right was considered healthy with a score of 1, and overeating or undereating was considered unhealthy with a score of 0. Current smoker was given a score of 0 and a nonsmoker was given 1. As a result, lifestyle had a total of 5 scores, from which 0–2 were considered as bad and 3–5 were considered as good lifestyle practice.

### 2.10 Statistical Analysis

Data were analyzed using IBM SPSS version 23. Socio demographic and lifestyle variables were calculated using descriptive statistics. The normality of the data was confirmed using the Shapiro‐Wilk and Kolmogorov‐Smirnov tests. Chi‐square tests of independence were employed to investigate the associations between socio‐demographic factors (e.g., age, gender, family type, BMI) and lifestyle variables. Statistical significance was considered as a *p*‐value < 0.05. Multivariate logistics regression models were applied to identify the independent predictors. Adjusted odds ratio with 95% confidence interval (CI) and *p* values are reported.

## 3 Results

Out of 215 total participants with the mean age of 18.69 years (standard deviation [SD] = 0.46), 75.8% (163) were females and 24.5% (52) were males. 70.2% (151) participants reported residence of urban area. The key socio‐demographic characteristics of the 215 students are summarized in Table 1. Regarding BMI distribution of participants 18.6% (*n* = 40), 68.8% (*n* = 148), 7.9% (*n* = 17), and 4.7% (*n* = 10) were underweight, normal, overweight, and obese. Of the 215 participants with complete lifestyle data, 120 (55.8%) demonstrated a good lifestyle score (3–5), while 95 (44.2%) had a poor lifestyle score (0–2).

**Table 1:** Basic socio‐demographic characteristics of participants (*n* = 215).

Variable		Mean ± SD	
Age (years)		18.69 ± 0.46	
		Frequency *n*	Percentage %
Age groups	18 years	67	31.2%
	19 years	148	68.8%
Gender	Male	52	24.5%
	Female	163	75.8%
Ethnic origin	Urdu‐speaking	114	53.0%
	Sindhi	21	9.8%
	Punjabi	44	20.5%
	Pathan	18	8.4%
	Balochi	1	0.5%
	Siraiki	17	7.9%
Residence	Urban	151	70.2%
	Rural	64	29.8%
Religion	Islam	213	99.1%
	Christianity	2	0.9%
Family type	Nuclear	169	78.6%
	Joint	46	21.4%
Monthly family income (PKR)	< 150, 000	51	23.7%
	90,000–149,999	37	17.2%
	60,000–89,999	70	32.6%
	40,000–59,999	41	19.1%
	10,000–24,999	7	3.3%
Father's education	No formal education	13	6.1%
	Primary (1–5)	4	1.9%
	Middle (6–8)	18	8.5%
	Matric (9–10)	29	13.7%
	Intermediate (11–12)	88	41.5%
	Bachelor's	26	12.3%
	Master's/Higher	34	16.0%
Mother's education	No formal education	27	12.7%
	Primary (1–5)	16	7.5%
	Middle (6–8)	24	11.4%
	Matric (9–10)	75	35.4%
	Intermediate (11–12)	21	9.9%
	Bachelor's	32	15.1%
	Master's/Higher	17	8.0%
Father's occupation	Laborer	15	7.0%
	Farmer	17	7.9%
	Business owner	24	11.2%
	Government employee	80	37.2%
	Private sector employee	42	19.5%
	Retired	37	17.2%
Mother's occupation	Housewife	193	89.8%
	Government employee	8	3.7%
	Private sector employee	14	6.5%
Lifestyle categories	Good lifestyle	120	55.8%
	Bad lifestyle	95	44.2%

### 3.1 Health and Lifestyle Practices

#### 3.1.1 Mental Health

Only 10.2 (*n* = 22) sought professional care, despite 28.4 (*n* = 61) reporting feelings of helplessness, worthlessness or helplessness as shown in Table 2.

**Table 2:** Lifestyle patterns (*n* = 215).

Section	Variable	Frequency *n*	Percentage %
Self‐reported feelings of hopelessness	Yes	61	28.4%
	No	154	71.6%
Night‐time activities	Sleeps < 8 h	64	24.8%
	Social media	91	35.3%
	Family time	44	17.1%
	Studying	16	6.2%
	Movies	5	1.9%
	Other combinations	49	18.3%
Physical exercise patterns	Exercises ≥ 3 times/week	29	13.5%
	Exercises < 3 times/week	25	11.6%
	Believes exercise essential but not regular	133	61.9%
	Exercises > 1 h/session	9	4.2%
	Believes exercise essential + regular	19	8.8%

#### 3.1.2 Dietary Patterns

**Figure 1:**
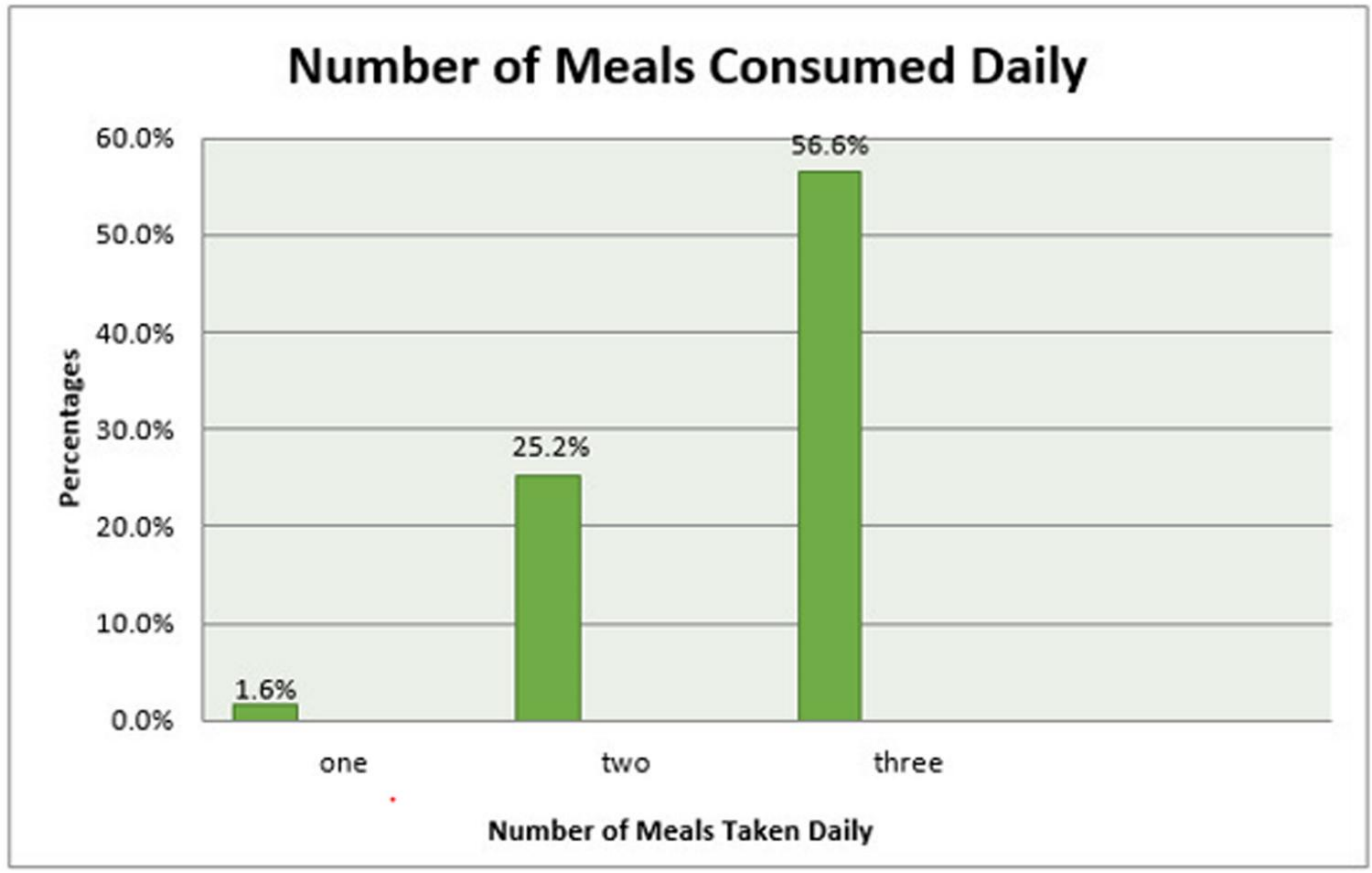
Number of meals taken daily (*n* = 215).

**Figure 2:**
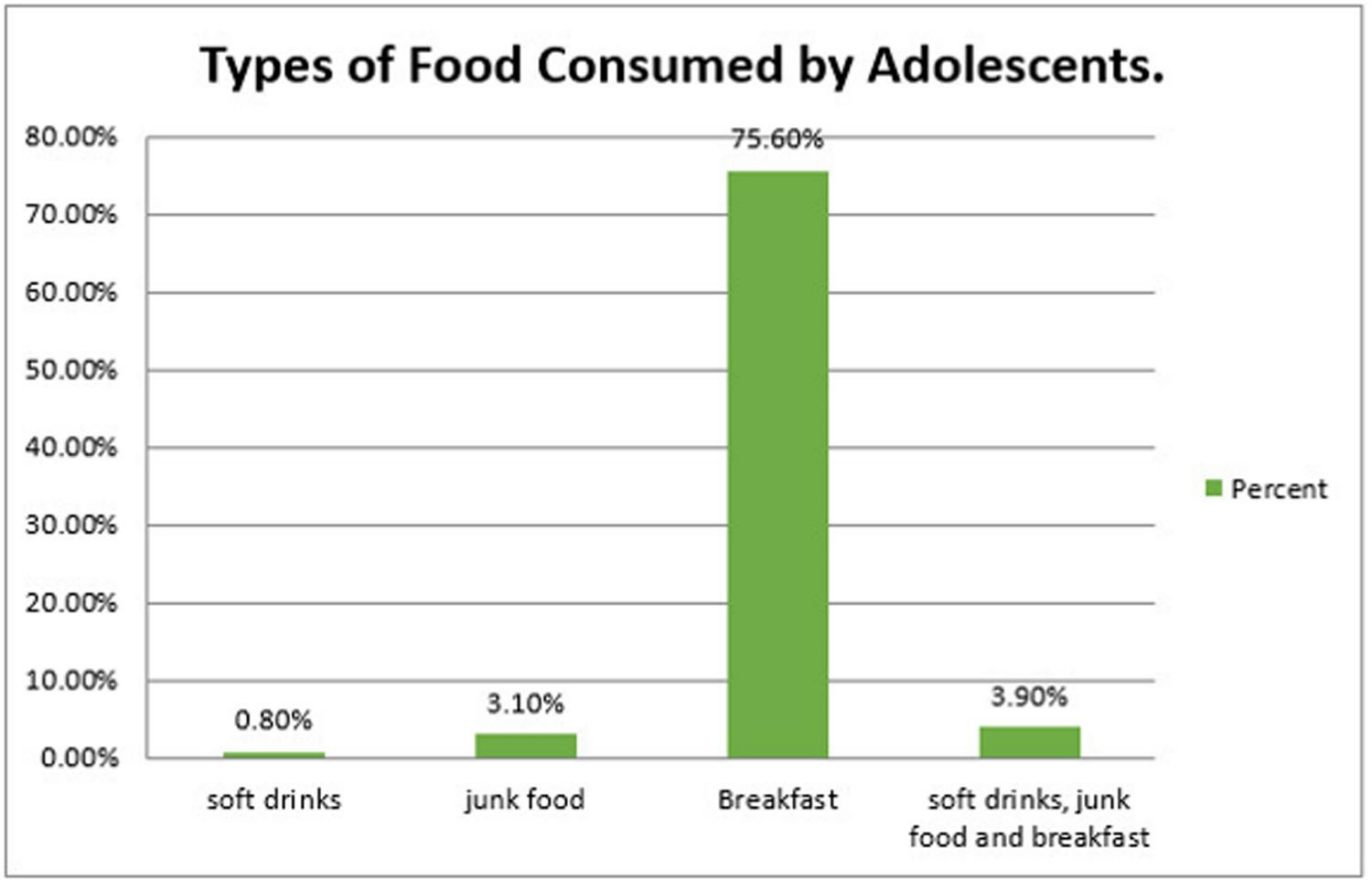
Type of food consumed (*n* = 215).

Nearly every adolescent (90.7 percent, *n* = 195), who regularly eats three main meals a day (Figure 1), reported adding junk food and soft drinks for breakfast (4.7%, *n* = 10), as well as a soft drink (0.9%, *n* = 2). Regarding portion size, 77.7% (*n* = 167) thought it was “just right,” 15.8% (*n* = 34) thought it was too small, and 6.5% (*n* = 14) thought it was too large (Figure 2). Stress eating was observed in 19.5% of the population (*n* = 42) as shown in Table 3.

**Table 3:** Health behaviours and lifestyle patterns among adolescents.

Category	Item	Percentage %
How many meals do you take daily	One	1.6%
	Two	25.2%
	Three	56.6%
Types of food consumed by adolescents	Soft drinks	0.80%
	Junk food	3.10%
	Breakfast	75.60%
	Soft drinks, junk food and breakfast	3.90%
Prevalence of cigarette smoking among adolescents	Yes	9.80%
	No	90.20%
Vaping status among adolescents	Yes	20.90%
	No	79.10%
Age at which adolescents started smoking and vaping (*n* = 215)	More than 12 years	74.4%
	Less than 12 years	25.6%
Awareness of adverse effects of smoking/vaping (*n* = 215)	Cancer	25.1%
	Lung disease	17.2%
	Cancer, lung diseases, heart diseases and other issues	41.4%
	Cancer and lung diseases	16.3%
Reasons of vaping and smoking (*n* = 215)	Peer pressure	23.3%
	Parent smokes	9.3%
	Get relief from stress	4.7%
	Gives confidence	9.3%
Weekly internet usage among adolescents (*n* = 215)	More than 40 h	9.3%
	20–40 h	32.6%
	Less than 20 h	58.1%

#### 3.1.3 Sleep and Bedtime Activities

As shown in Table 2, sleeping for less than 8 h was reported by 24.8 percent (*n* = 64); bedtime < 12 midnights: 45.3 percent (*n* = 117); after 12 midnights was reported by 13.2 percent (*n* = 34) of adolescents. In the evenings, people used social media (35.3%, *n* = 91), spent time with their families (17.1%, *n* = 44), studied (6.2%, *n* = 16), watched movies (1.9%, *n* = 5), and did other things (18.3%, *n* = 49).

#### 3.1.4 Physical Exercise

While 61.9% of university‐going adolescents thought exercise was beneficial for their health, only 11.6% exercised on a weekly basis, and only 4.2% attended programs that lasted more than an hour (Table 2).

#### 3.1.5 Substance Use and Awareness

**Figure 3:**
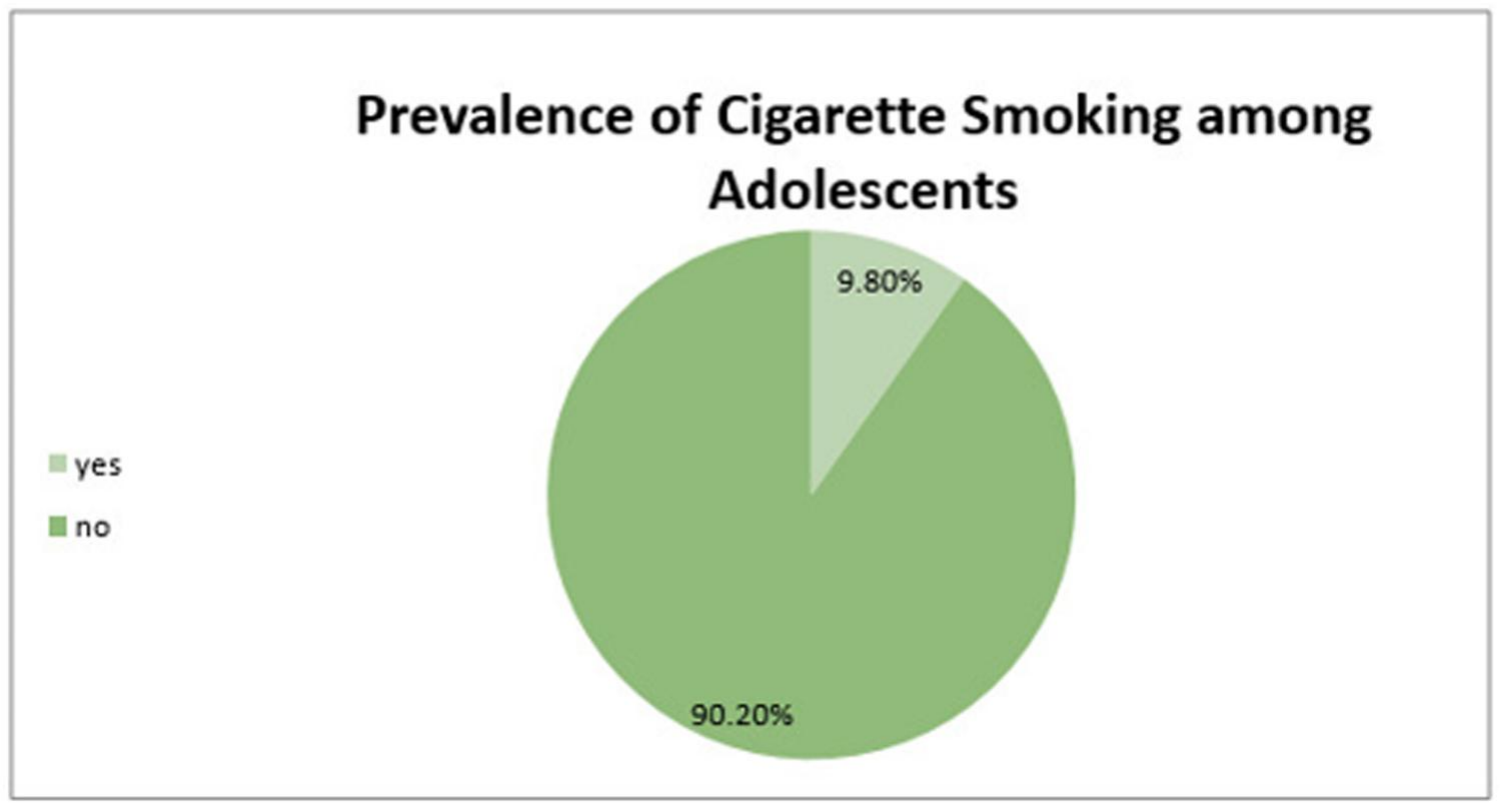
Prevalence of cigarette smoking among adolescents (*n* = 215).

**Figure 4:**
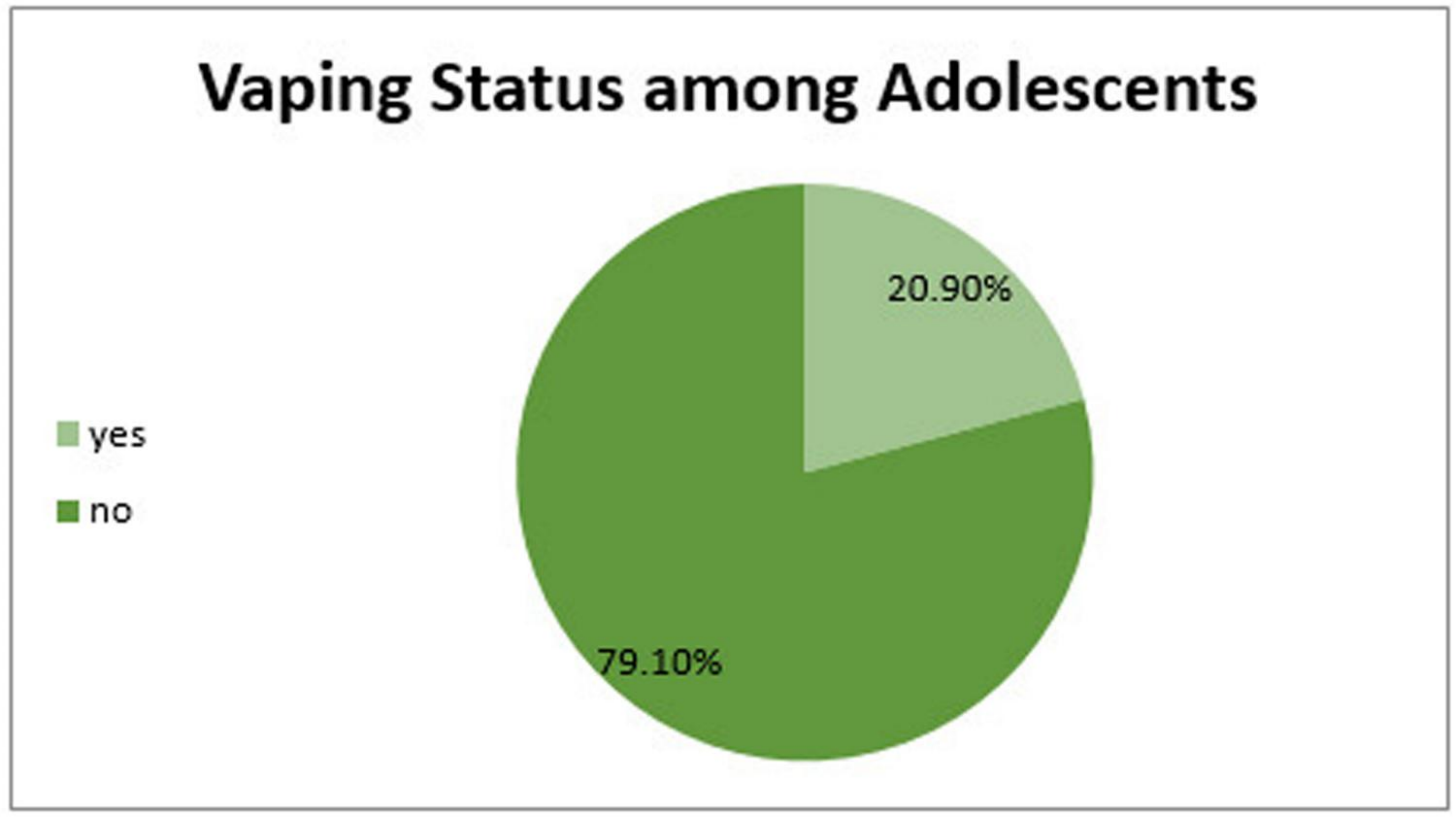
Vaping status among adolescents (*n* = 215).

**Figure 5:**
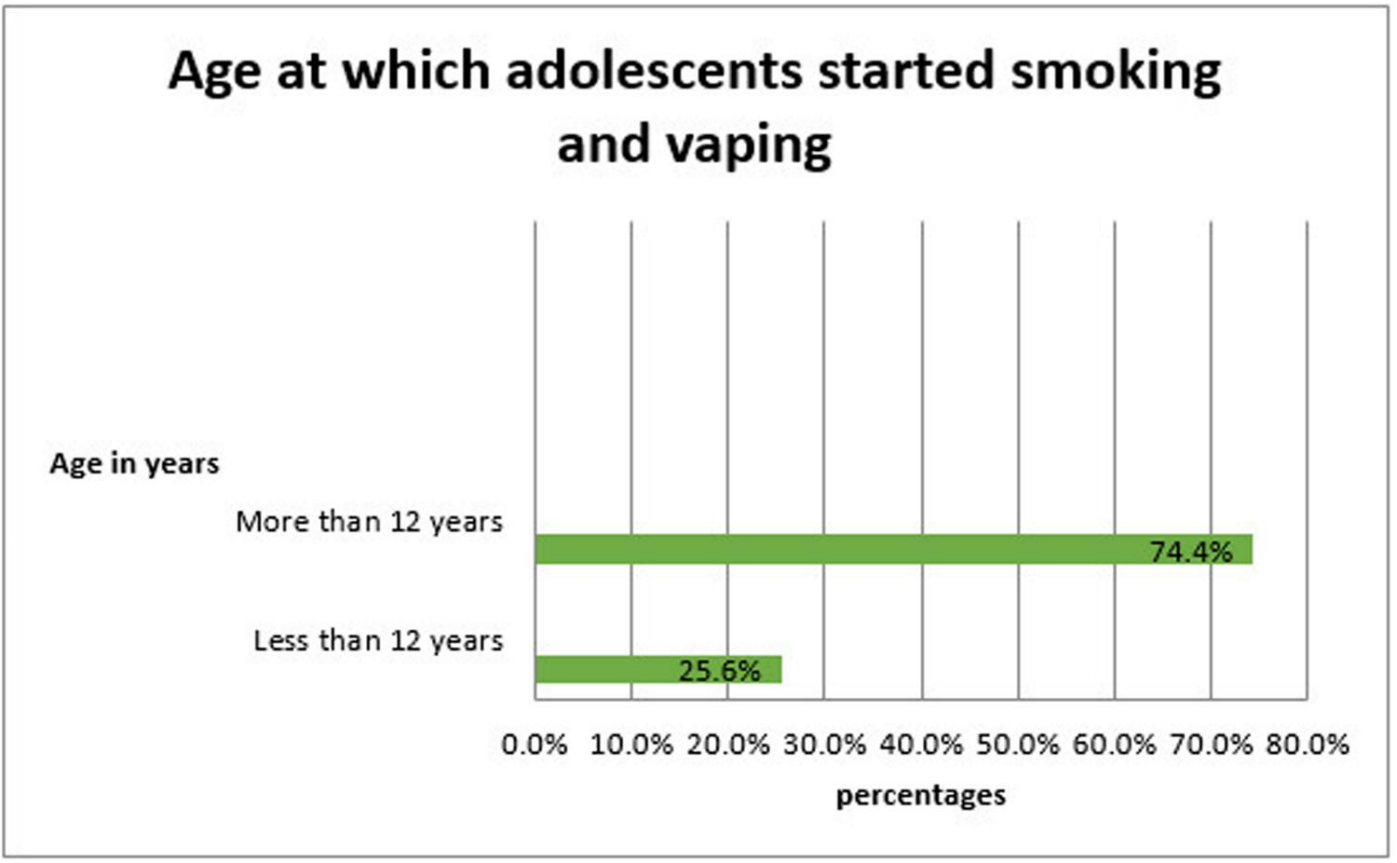
Age at which adolescents started smoking and vaping (*n* = 215).

**Figure 6:**
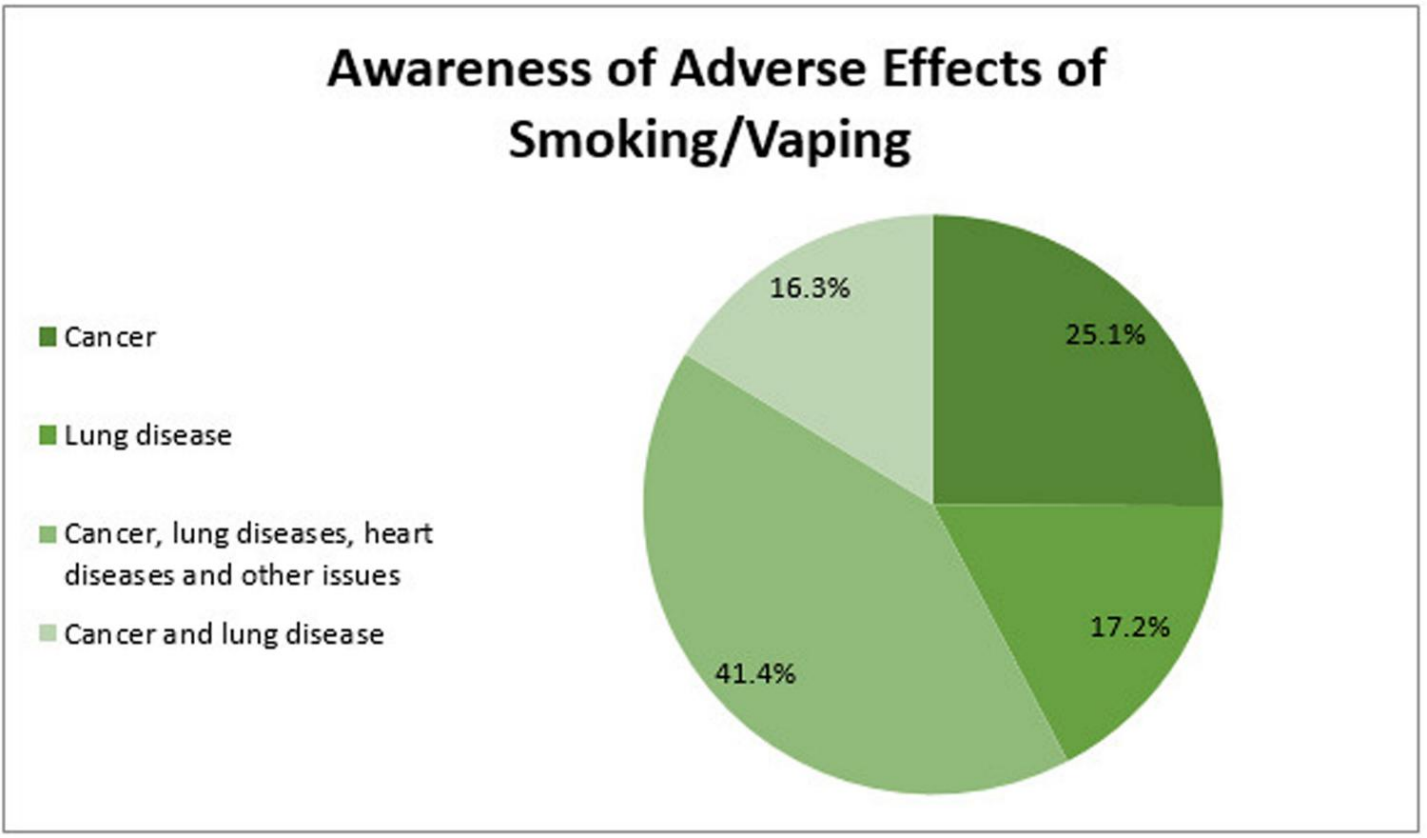
Awareness of adverse effects of smoking/vaping (*n* = 215).

**Figure 7:**
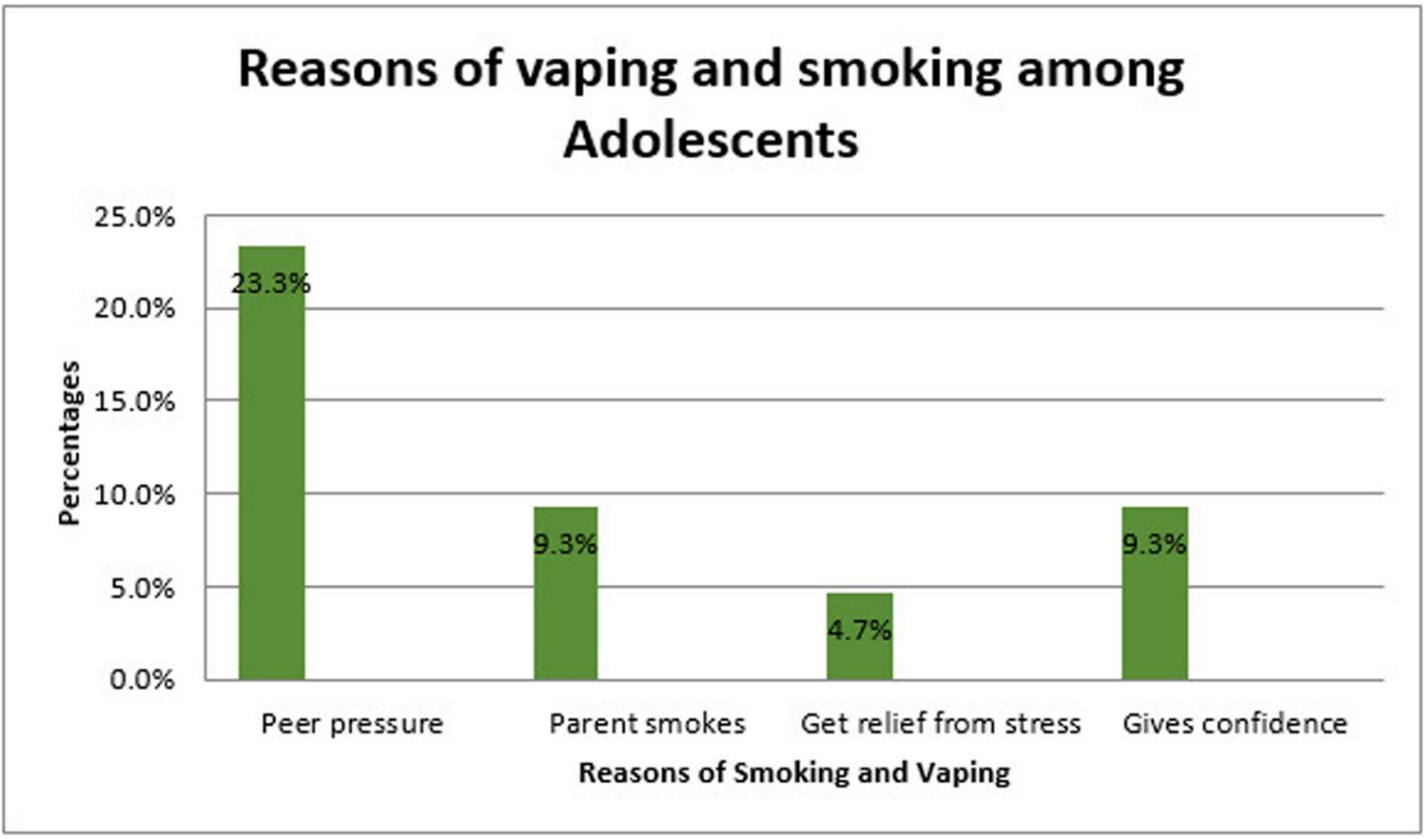
Reasons of vaping and smoking (*n* = 215).

10.2% of adolescents (*n* = 22) reported using sleeping drugs. 9.8% of respondents (*n* = 21) said they smoked cigarettes (Figure 3), 20.9 percent (*n* = 45) have vaped (Table 3, Figure 4). 25.6% (*n* = 11) had an early onset (< 12 y), while 74.4% (*n* = 32) had a later onset (Table 3, Figure 5). Smoking and vaping was reported predominantly among males; 32 percent of males (17/52) smoked, whereas in females it was 2.4% (4 out of 163). 41.4% of respondents were aware of several dangers, 25.0% were aware of cancer as a risk factor alone, 17.2% were aware of lung disease alone, and 16.3% were aware of both cancer and lung disease, as shown in Table 3 and Figure 6. The most common reason reported for smoking or vaping was peer pressure, accounting for 23.3% of responses. Both parental smoking and the belief that it boosts confidence were cited by 9.3% of adolescents. A smaller proportion (4.7%) indicated they smoke or vape to relieve stress as shown in Figure 7. These findings suggest that social influence and perceived psychological benefits play a significant role in adolescents' decision to initiate smoking or vaping, as shown in Table 3.

#### 3.1.6 Body Image and Internet Usage

**Figure 8:**
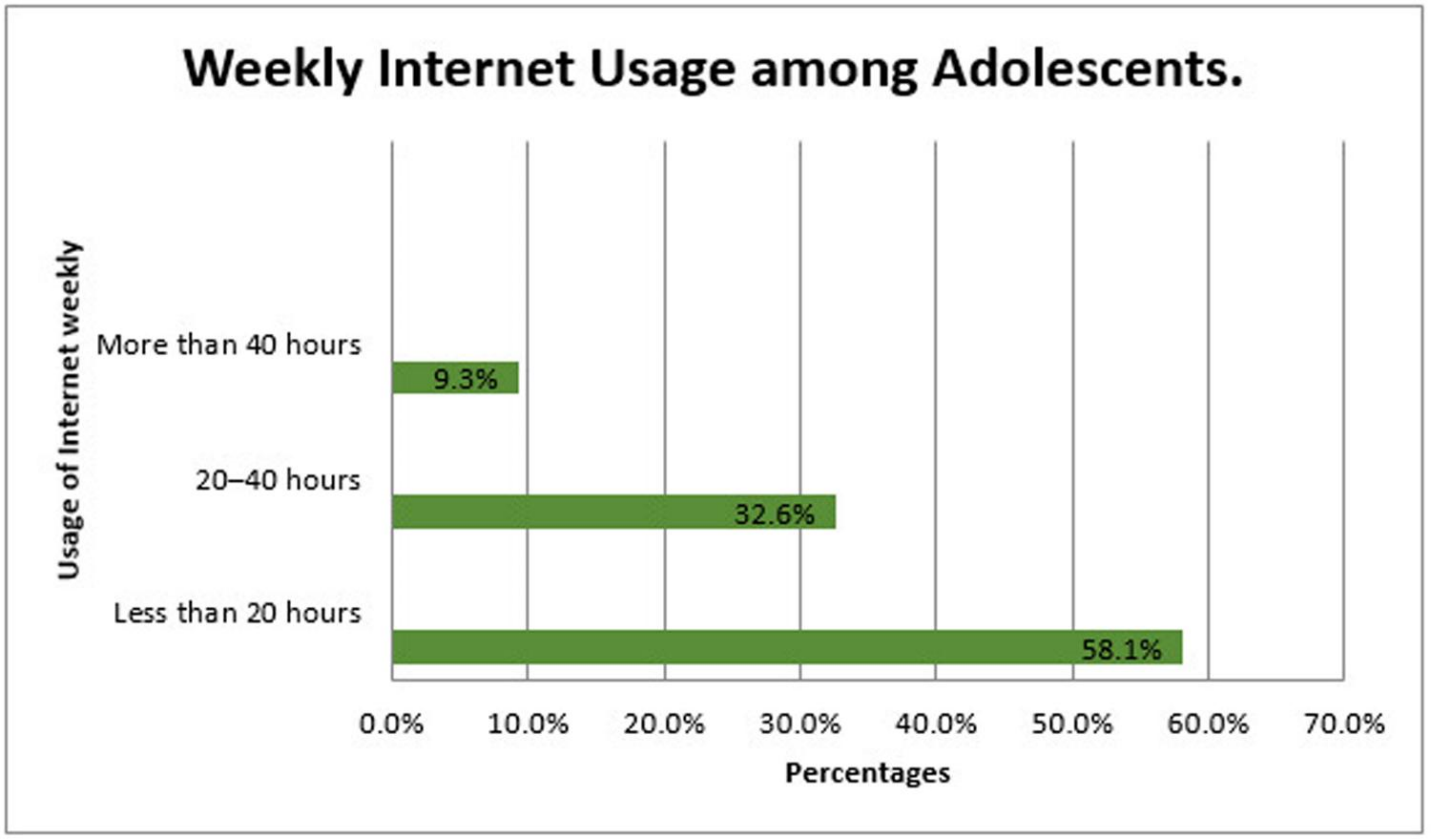
Weekly internet usage among adolescents (*n* = 215).

38 percent (*n* = 82) said they were insecure about their bodies. 31.6% of people choose to stream drama; they also frequently watch science, music, and sports shows. Weekly internet usage: < 20 h (58%), 2040 h (32%), and 40+ hours (9%) as shown in Table 3 and Figure 8.

### 3.2 Associations Between Lifestyle Variables and Socio‐Demographic Characteristics

Using chi‐square analyses, a number of significant relationships between the main lifestyle variables and socio‐demographic factors were found. There was no difference in the lifestyle profile of male and female students, since gender did not correspond with any of the factors related to health complaints, eating patterns, mental health indicators, sleep practices, and substance use culture. In contrast, there were significant differences between the 18 and 19‐year‐old age groups; older adolescents had a wider range of health complaints (chi‐square = 47.680, df = 13, *p* < 0.001), different meal frequencies (chi‐square = 15.888, df = 2, *p* < 0.001); shifted to later bedtimes (chi square = 6.934, df = 2, *p* = 0.031), and late‐night activities (chi‐square = 42.949, df = 10, *p* < 0.001). There was no significant association found between body image concerns and socio‐demographic variables. However weekly internet usage was found to be significantly associated with participant age, older adolescents were more likely to report higher internet usage as shown in Table 4.

**Table 4:** Multivariable associations and regression analyses of lifestyle factors.

Predictor	Linear B (95% CI)	Linear β	Linear *p*‐value	Logistic AOR (95% CI)	Logistic *p*‐value
Gender	−0.281 (−0.641 to 0.079)	−0.121	0.126	0.622 (0.239–1.617)	0.330
Age	0.156 (−0.134 to 0.447)	0.072	0.290	1.352 (0.628–2.912)	0.440
Ethnic origin	0.077 (−0.023 to 0.177)	0.114	0.131	1.148 (0.846–1.557)	0.376
BMI	−0.233 (−0.437 to −0.029)	−0.156	0.025	0.496 (0.270–0.911)	0.024
Urban residence	0.635 (0.346 to 0.923)	0.293	< 0.001	12.021 (4.706–30.708)	< 0.001
Father's education	0.188 (0.070 to 0.306)	0.290	0.002	1.899 (1.296–2.783)	0.001
Mother's education	−0.086 (−0.199 to 0.028)	−0.149	0.138	0.712 (0.522–0.971)	0.032
Family income	0.032 (−0.076 to 0.140)	0.042	0.557	0.982 (0.733–1.317)	0.906
Joint family	−0.496 (−0.811 to −0.181)	−0.205	0.002	0.300 (0.125–0.717)	0.007
Smoking	0.340 (−0.127 to 0.807)	0.102	0.152	3.740 (0.947–14.776)	0.060

*Note:* Linear model: F(10, 201) = 6.069, *p* < 0.001; R = 0.482; R^2^ = 0.232; Adjusted R^2^ = 0.194. Logistic model: Omnibus χ^2^ = 71.587, df = 10, *p* < 0.001; Cox and Snell R^2^ = 0.287; Nagelkerke R^2^ = 0.383; Hosmer‐Lemeshow χ^2^ = 37.825, df = 8, *p* < 0.001; classification accuracy = 74.5%.

Eating habits (chi square = 7.320, df = 2, *p* = 0.026), and physical exercise (*p* < 0.0001) were all significantly correlated with family type. Stress eating, feelings of helplessness, worthlessness or helplessness, daily meal count (*p* < 0.098), and current smoking/vaping were not significant (Table 3). BMI categories were closely associated with frequency of physical activities (chi‐square = 75.736, df = 12, *p* < 0.001), eating patterns (chi‐square = 46.597, df = 6, *p* < 0.001), and health complaints (chi‐square = 133.923, df = 39, *p* < 0.001). These findings demonstrate the subtle ways in which university‐going older adolescents socio‐demographic circumstances influence their way of life as shown in Table 4.

### 3.3 Regression Analysis of Lifestyle Determinants

In order to comprehend the determinants that shape the participants' lifestyle, both linear and logistic regression analyses were conducted using the same ten socio‐demographic and behavioral predictors: gender, age, ethnicity, BMI, residency, paternal education, maternal education, family income, family structure, and smoking status (Table 4).

The linear regression model was statistically significant overall (F(10, 201) = 6.069, *p* < 0.001) and accounted for 19.4% of the variance in lifestyle score (Adjusted R^2^ = 0.194). Significant predictors of a higher (more positive) lifestyle score were lower BMI (β = −0.156, *p* = 0.025), urban residence (β = 0.293, *p* < 0.001), and higher paternal education (β = 0.290, *p* = 0.002), while joint family structure was associated with a lower lifestyle score (β = −0.205, *p* = 0.002). Maternal education, family income, gender, age, ethnicity, and smoking status were not statistically significant predictors in this model.

The logistic regression model was also significant (χ^2^ = 71.59, df = 10, *p* < 0.001), correctly classifying 74.5% of cases and explaining 28.7% (Cox and Snell R^2^) to 38.3% (Nagelkerke R^2^) of the variance in lifestyle category. Adjusted odds of good lifestyle were significantly higher for participants with lower BMI (AOR = 0.50, 95% CI: 0.27–0.91, *p* = 0.024), urban residence (AOR = 12.02, 95% CI: 4.71–30.71, *p* < 0.001), higher paternal education (AOR = 1.90, 95% CI: 1.30–2.78, *p* = 0.001), and higher maternal education (AOR = 0.71, 95% CI: 0.52–0.97, *p* = 0.032), while joint family structure was associated with significantly lower odds of good lifestyle (AOR = 0.30, 95% CI: 0.13–0.72, *p* = 0.007). Smoking approached but did not reach statistical significance (AOR = 3.74, 95% CI: 0.95–14.78, *p* = 0.060).

## 4 Discussion

The study addresses significant issues regarding the health and lifestyle choices of University‐Going Adolescents enrolled in a public university in Rawalpindi. Regular family time and eating breakfast are two important healthy habits, but there are still some problems. Key issues are thoroughly examined, connected to previous research, and attempt to provide clarifications.

Recent research conducted within the country has pointed out lifestyle challenges pertaining to the young population in Pakistan. To illustrate, one study conducted with medical students found that during the COVID‐19 lockdown, 48% of students were physically inactive and 45% students exhibited high levels of sedentary behavior [15]. Considering how the present study found that less than 12% of the participants met the recommended levels of physical activity, this points to an even greater risk amongst older adolescents attending university. Likewise, research based in schools has documented the relationships between the socio‐demographic factors of younger adolescents (such as socio‐economic status and geo location) and the amount of physical activity undertaken [16].

Turning to substance use behaviors, comparative data from other Pakistani university populations provide useful context a study on university students in Pakistan documented that 46.6% of the students were involved in smoking and/or vaping [17]. Another study conducted in Karachi reported that 50.4% of college students were vapers [18]. Although present sample reported a vaping prevalence of 20.9% with vaping initiation occurring below 12 years of age which is lower than the vaping prevalence documented in other studies, it is still high, which points out that older adolescents who attend university remain a high‐risk population. Overall, these findings verify that this study population is concerned with research that is relevant to the age, however, the studies participants attend university demonstrates that they are exposed to several risk factors that are heightened.

Dietary patterns represent another critical lifestyle domain in this population in comparison to the present study results in Spain (13.7%–19.4%), the United States (27% in 2023), and Denmark (37%), almost all students in Ireland (90.7%) eat breakfast every day [19]. Even though most participants felt their food quantities were sufficient, the HELENA data reveals that individuals who felt they ate less might have a higher BMI [20]. Studies involving German and Dutch teenagers likewise found a correlation between stress and increased food consumption [14], as did Swedish overweight boys (odds ratio > 1.5) [21], Another study found warning indications of internet addiction include weight loss or gain, a decline in academic performance, and skipping meals, activities, and assignments for social media [22].

In our sample, 97 percent of respondents agreed that they should eat three meals a day, and harmful behaviors such as overeating or adding junk food to meals were also noted (19.5% of respondents merely stated that they ate because they were stressed). Present study validates a Rawalpindi observation from 2022 that the adolescent population's food patterns had changed negatively, posing a significant risk of developing non‐communicable diseases [23, 24] as harmful behaviors such as overeating, or junk food consumption was also reported by our participants of Rawalpindi.

Mental health indicators also warrant discussion alongside these behavioral findings the present study reported adolescents experiencing feelings of hopelessness, worthlessness, or helplessness in the study setting. In comparison, depressive symptoms were reported by nearly one in three Canadian participants (28.4%), which is comparable to the prevalence among Chinese and global teenagers and has been rising since COVID. Significant portions of Bulgarian youth (10.2%) share this difficulty with youth around the globe: they are uncomfortable using health care services, (UNICEF, 2025). Similarly, another study found that the prevalence of raised depressive symptoms is highest in the Middle East, Africa, and Asia, and it is higher among female teenagers than male adolescents and Between 2001 and 2010, the point prevalence of high depressive symptoms in teenagers was 24%; between 2011 and 2020, it rose to 37% [25].

Hydration and sleep patterns showed similarly mixed results relative to international benchmarks a Canadian study found that 55%–65% of adults get the required quantity of water after COVID‐19 [26] indicating that most people are learning about water consumption, even while 47% of adults don't drink enough water each day. People who use screens and may have sleep issues are similar to those in international research [27], who sleep for less than 8 h (29.8%) and go to bed after midnight (54.4%). The same report by the WHO states that only 81% of adolescents globally are regularly active, which matches the findings here [28, 29]. The percentage of physically inactive Chinese adolescents jumped from 21.3% to 65.6% during the pandemic. 19% of adolescents in the present study experienced bullying, which is lower than UNESCO's reported range of 25% to 30% victimization rates found elsewhere. Also, 32% of those surveyed said they use the internet and social media for very long periods every week, which fits with research connecting frequent screen time to possible eating disorders and difficulties sleeping in children and teenagers (Noris et al., 2022). aping and early initiation before age 12 in our sample greatly outstrip normal U.S. high school rates (10%); our own vaping prevalence of 20.9% is itself notably high for a university‐attending population, consistent with rising vaping trends reported elsewhere [30].

Present study identified a substantial relationship between health habits and residence, age, and BMI. Living in an urban region was highly linked to consuming more junk food, using the internet, and spending more time on screens during free time. This is also the case in Karachi, where urban youths were found to consume more soft drinks and engage in less physical activity [25]. Qidwai and his colleagues' Karachi research, conducted 15 years ago, found that 58.9% of participants did not get enough sleep, many picked exercise last, and smoking was common when persuaded by others. Concerning our Rawalpindi sample, we can see some changes in behaviors (more balanced breakfasts; attitudes on stress management), but there are still concerns with depression and substance addiction [14].

Strengths of the study are that it provides recent and direct data on lifestyle behaviors of university going older adolescents in Rawalpindi, Pakistan. However, the study has some limitations; it is unable to establish causality. As the sample solely includes students from single public university of Rawalpindi, use of purposive sampling and over representation of female and urban population the results cannot be generalized. This study followed STROBE guidelines for cross‐sectional reporting where applicable; however, the absence of a directly comparable lifestyle‐prevalence estimate for sample size calculation, as noted in Methods, is acknowledged as a limitation. Furthermore, participants may have provided biased responses since all data was based on self‐reported data, which may be subjected to recall and social desirability bias. Some measures were single‐item and lacked full psychometric validation in present setting (they were pilot‐tested for face validity). Future studies should use probability sampling across multiple institutions.

## 5 Conclusion

University‐going older adolescents in Rawalpindi engage in both protective and risky health behaviors: while breakfast consumption and family time were common, physical activity, hydration, and sleep were frequently inadequate, and nearly a third reported depressive symptoms with few seeking professional care. Health behaviors were consistently associated with age, BMI, parental education, and family structure. These findings support the need for targeted university‐based interventions addressing mental health, substance use prevention, and physical activity promotion among this population.

## Author Contributions

**Meesha Rao:** conceptualization, methodology, data curation, writing – original draft, formal analysis. **Humaira Mahmood:** conceptualization, methodology, formal analysis, writing – original draft, supervision. **Abdul Momin Rizwan Ahmad:** writing – review and editing, methodology, formal analysis. **Saira Zafar:** conceptualization, writing – original draft. **Hasnain Khalid:** conceptualization, data curation, writing – review and editing, formal analysis. **Manaar Adil Siddiqui:** methodology, data curation, writing – review and editing, formal analysis. **Naseem Azad:** conceptualization, writing – review and editing. **Attiya Najeeb Abbasi:** conceptualization, writing – review and editing, formal analysis. **Nilab Mohammadi:** methodology, writing – review and editing.

## Funding

The authors have nothing to report.

## Ethics Statement

Ethical approval for this study was obtained from the Ethical Review Committee of the Armed Forces Postgraduate Medical Institute (AFPGMI), Rawalpindi vide letter no. 535‐AAA‐ERC‐AFPGMI. Moreover, written informed consent was obtained from all participants prior to enrollment. Only those were included who agreed to participate. All the research protocols were carried out in accordance with the Declaration of Helsinki.

## Conflicts of Interest

The authors declare that they do not have any conflict of interest.

## Transparency Statement

The corresponding author (Nilab Mohammadi) affirms that this article is an honest, accurate, and transparent account of the study being reported; that no important aspects of the study have been omitted; and that any discrepancies from the study as planned (and, if relevant, registered) have been explained.

## Data Availability

The data that support the findings of this study are available from the corresponding author upon reasonable request. The raw data supporting the conclusions of this article will be made available by the authors, without undue reservation.
